# A Plant-Feeding Nematode Indirectly Increases the Fitness of an Aphid

**DOI:** 10.3389/fpls.2017.01897

**Published:** 2017-11-03

**Authors:** Grace A. Hoysted, Catherine J. Lilley, Katie J. Field, Michael Dickinson, Sue E. Hartley, Peter E. Urwin

**Affiliations:** ^1^Centre for Plant Sciences, University of Leeds, Leeds, United Kingdom; ^2^FERA Science Ltd., York, United Kingdom; ^3^Department of Biology, University of York, York, United Kingdom

**Keywords:** aboveground–belowground interactions, aphids, induced defenses, jasmonic acid, plant parasitic nematodes, salicylic acid

## Abstract

Plants suffer multiple, simultaneous assaults from above and below ground. In the laboratory, pests and/or pathogen attack are commonly studied on an individual basis. The molecular response of the plant to attack from multiple organisms and the interaction of different defense pathways is unclear. The inducible systemic responses of the potato (*Solanum tuberosum* L.) host plant were analyzed to characterize the plant-mediated indirect interactions between a sedentary, endoparasitic nematode (*Globodera pallida*), and a phloem-sucking herbivore (*Myzus persicae*). The reproductive success of *M. persicae* was greater on potato plants pre-infected with *G. pallida* compared to control plants. Salicylic acid (SA) increased systemically in the leaves of potato plants following nematode and aphid infection singly with a corresponding increase in expression of SA-mediated marker genes. An increase in jasmonic acid associated with aphid infection was suppressed when plants were co-infected with nematodes. Our data suggests a positive, asymmetric interaction between a sedentary endoparasitic nematode and a sap-sucking insect. The systemic response of the potato plant following infection with *G. pallida* indirectly influences the performance of *M. persicae*. This work reveals additional secondary benefits of controlling individual crop pests.

## Introduction

Plants are simultaneously attacked by a number of invading organisms, both above and below ground. Pests and pathogens sharing the same host can, despite their spatial separation, together elicit a response that is more complex than the additive response of those sole agents (van Dam and Heil, 2011). Infection of a host plant that carries a pre-existing pest or pathogen burden will influence the success of the secondary or primary infection, depending on a range of factors including the species under investigation, the sequence of pest arrival, the severity of the infestation (Erb et al., 2011; Johnson et al., 2012; Huang et al., 2016; Papadopoulou and van Dam, 2017), and the changes in primary and secondary metabolites in the shared plant tissues (Bezemer et al., 2003; Wardle et al., 2004; Schoonhoven et al., 2005; van Geem et al., 2016). Given this context dependency, it is unsurprising that both positive and negative effects of below-ground organisms on those above-ground have been reported. For example, a positive indirect influence by generalist root herbivores resulted in an increased abundance of a tephritid (Diptera: Tephritidae) seed predator and two of its dominant parasitoids (Hymenoptera: Chalcidoidea) on the marsh thistle (Masters et al., 2001), whereas negative indirect effects of wireworms below ground led to a reduced performance and fecundity of the beet armyworm, a major foliage feeding pest of cotton (Bezemer et al., 2003).

Host-mediated interactions between plant-feeding organisms are particularly significant in agricultural systems: many economically important crops are attacked simultaneously by aboveground insect pests, such as aphids, and by belowground pathogens, such as plant parasitic nematodes. Aphids, the largest group of phloem feeders, use their stylet-like mouthparts to feed on photoassimilates found in the phloem sieve elements (Pollard, 1973). Aphids also transmit viruses, which can adversely affect the fitness of the host plant (Dixon and Kindlmann, 1998). Primarily, their importance is as vectors of virus diseases but due to their ability to reproduce rapidly (Foster et al., 2000), high populations can also result in substantial reductions in yield (Kolbe, 1970). Cyst nematodes are a group of highly evolved sedentary endoparasites and are pathogens of temperate, subtropical and tropical plant species. Following root penetration, cyst nematode second-stage juveniles migrate intracellularly toward the vascular cylinder where each chooses an initial syncytial cell from which it will form a highly metabolically active feeding site (Lilley et al., 2005). Large scale gene expression profiling has identified genes that are differentially regulated by cyst nematode infection following a compatible interaction (Alkharouf et al., 2006; Ithal et al., 2007; Szakasits et al., 2009) and many genes related to metabolic pathways including phytohormone regulation are up-regulated in the host plant (Uehara et al., 2010). Salicylic acid (SA)-dependent signaling seems to be crucial for resistance against biotrophic pathogens (Glazebrook, 2005; Loake and Grant, 2007) and cyst nematodes have been reported to activate a strong SA-mediated defense response in shoots of *Arabidopsis thaliana* from 5 days post inoculation (Wubben et al., 2008).

Although cyst nematodes and aphids may share the same host, their infection of the plant is temporally as well as spatially separated: nematodes infect plants soon after roots emerge, while aphids colonize plants later in the year, once there is sufficient biomass above ground (Van Emden et al., 1969). This temporal separation may give rise to asymmetric interactions, whereby nematodes influence the performance of aphids, but aphids do not impact on nematodes. There is some evidence to support this in that there are more studies demonstrating that nematodes have an effect on the performance and fecundity of aphids than *vice versa* (Kutyniok and Müller, 2012). The mechanism underpinning this asymmetric interaction may be changes to plant biomass, although changes in primary and secondary metabolites appear to be more important at least in some cases. For example, a mixed nematode infection of *Pratylenchus, Meloidogyne*, and *Heterodera* spp. has been reported to reduce the fecundity of *Schizaphis rufula* without significantly affecting plant biomass (Vandegehuchte et al., 2010). Similarly, an increase in phenolic content in foliar parts of plants has been reported following infection with plant parasitic nematodes (van Dam et al., 2005; Kaplan et al., 2008), which had a negative effect on the survival rate of above-ground herbivores. In a study of interactions between the soybean aphid and the soybean cyst nematode, alate aphids preferred plants without nematodes over nematode-infested plants, though the performance and population growth of aphids feeding on nematode-infested plants was either unaffected or even slightly improved (Hong et al., 2010). Systemic changes to primary and secondary metabolites have been reported in *Arabidopsis thaliana* infected with the beet-cyst nematode *Heterodera schachtii* (Hofmann et al., 2010). A similar response to *H. schachtii* in *Brassica oleracea* was subsequently reported to cause reduced aphid population growth and disturbed feeding relations between plants and aphids (Hol et al., 2013).

Phytohormones such as SA, jasmonic acid (JA), and ethylene (ET) are, or are at least partly, shared by both abiotic and biotic stress signaling, indicating the likelihood of crosstalk and convergence of mechanisms in these molecular pathways. Research aimed at developing stress-tolerant crops is therefore increasingly focussing on crosstalk between phytohormones (Miller et al., 2010; Denancé et al., 2013; Kissoudis et al., 2014). Crosstalk between different molecular signals is a way in which plants can fine-tune their responses to stress by controlling gene expression (Pieterse et al., 2012; Lazebnik et al., 2014). Phytohormones can act either at their site of synthesis or systemically elsewhere in the plant (Peleg and Blumwald, 2011), thus attack from a pathogen at one position in a plant may indirectly affect a secondary arriving pest through plant-mediated interactions. Complex interactions between SA, JA, and ET, however, are influenced by the invading pest or pathogen and the timing of the infection (Dicke et al., 2009; Ton et al., 2009; Atkinson et al., 2015).

In this study, we examined plant-mediated interactions between the plant parasitic nematode, *Globodera pallida* and the generalist aphid *Myzus persicae* Sulzer (Hemiptera: Aphididae) in the potato crop (*Solanum tuberosum* cv. Désirée). The potato cyst nematode *G. pallida* is an important pathogen of potato crops that can cause reported yield losses in excess of 50% (Trudgill, 1986) and the species is estimated to be present in 64% of potato-growing fields in England and Wales (Minnis et al., 2002). *M. persicae* feeds on a large variety of plants belonging to different families and worldwide is the most important insect pest of potato (Radcliffe, 1982). Although there is an increasing number of studies on nematode–aphid interactions in the model species *Arabidopsis thaliana* (Kutyniok and Müller, 2012; Kutyniok et al., 2014), the plant-mediated mechanisms responsible for such effects at both the biochemical and molecular level remain unexplored in crop plants. Using a combination of molecular and biochemical techniques, we test the hypothesis that systemic changes in endogenous phytohormones and the expression of associated genes can indirectly influence these plant-mediated interactions between organisms feeding above and below ground. We examine the induced systemic defense response of potato plants following nematode infection and how these responses impact on aphid-induced SA production which is required for systemic acquired resistance (SAR), leading to the expression of *PR-*genes. We also describe levels of endogenous JA and the expression of a gene involved in jasmonate signaling. Finally, we show the impact of *G. pallida* pre-infection of potato plants on *M. persicae* abundance.

## Materials and Methods

### Aphids and Nematodes

Nymphs of the peach-potato aphid (*Myzus persicae*) were obtained from the James Hutton Institute, Invergowrie, Dundee, Scotland. The aphids were asexual clones of a wild population isolated in Scotland (Kasprowicz et al., 2008). Aphid colonies were maintained on potato plants (*S. tuberosum* L. cv. Désirée) inside a mesh cage in a containment glasshouse at 20–22°C under a 16 h/8 h light/dark cycle. Only apterous (wingless) aphids were used and transferred to experimental plants using a fine paintbrush.

Cysts of *G. pallida* were extracted from infected soil stocks using the Fenwick can method (Fenwick, 1940). Infective second-stage juveniles (J2s) were hatched from the cysts following treatment with 1% sodium hypochlorite aqueous solution (Heungens et al., 1996). J2 nematodes were stored in autoclaved tap water at 10°C and their viability was checked prior to use by observation using a stereo binocular microscope.

### Pest and Pathogen Infection and Sample Collection

Potato tuber cuttings (*S. tuberosum* L. cv. Désirée) were planted in 18 cm pots containing pesticide-free compost. Growth took place in a glasshouse at 20–22°C under a 16 h/8 h light/dark cycle for a period of 3 weeks. For potato plants infected with nematodes only, 10,000 J2 nematodes suspended in 6 mL of autoclaved tap water were introduced into the compost around the roots of each potato plant. Uninfected potato plants used as a control were mock-inoculated with autoclaved tap water. At 14 days post inoculation (dpi), a fully expanded terminal leaf from the top of each plant was excised using fine tweezers, divided into three samples for RNA, SA, and JA extractions and immediately snap frozen in liquid nitrogen. Five-week-old potato plants were used for infection with aphids alone so ensuring each set of experimental plants were the same age. Twenty apterous aphids of various life-stages were transferred to the second fully expanded leaf with a fine paintbrush and confined to the abaxial surface of the leaf in a 2.5 cm diameter clip-cage. Aphid-free clip-cages were used in control experiments. After 48 h, aphids were carefully removed and the leaf was excised and sampled as previously described. Co-infected potato plants were initially inoculated with ten thousand J2 nematodes, then 14 days later 20 apterous aphids were applied to either infected or control plants for 48 h as previously described. Co-infected samples were collected 48 h post infection (hpi) with aphids.

### RNA Extraction, cDNA Synthesis, and qRT-PCR for the Analysis of *PR*-Gene Expression

Total RNA was prepared from frozen leaf tissue of control and infected potato plants using the RNeasy^®^ Plant Mini Kit (Qiagen, Inc., Valencia, CA, United States). First-strand cDNA was synthesized from 1000 ng RNA using SuperScript II reverse transcriptase (Invitrogen, Carlsbad, CA, United States) and oligo(dT)_17_ primer (500 μg/ml) following the manufacturer’s instructions. Quantitative reverse transcriptase (qRT)-PCR was carried out on the resulting cDNA using Brilliant III Ultra-Fast SYBR^®^ Green Master Mix and a Mx3005P (v. 4.10) instrument (Agilent Technologies, La Jolla, CA, United States). Genes for expression analysis were selected according to their previously recorded involvement in biotic stress responses (Kombrink et al., 1988; Matton and Brisson, 1989; Fidantsef et al., 1999; Reiss and Horstmann, 2001; Wang et al., 2005) (see Results section for further details). Potato *ELONGATION FACTOR 1*-α was used to normalize the results (Nicot et al., 2005). Sequences of primers used for amplification of each gene are detailed in Supplementary Data Table 1. Sequences for the chosen genes were found on the National Center for Biotechnology Information website^1^ and primers were designed using the online Primer 3 software^2^. Controls for qRT-PCR included reactions containing no template. All primer pairs had an amplification efficiency of 93–101% and *R*^2^ correlation coefficients for standard curves ranged between 0.94 and 0.99. qRT-PCR was performed on five biological replicates for control and infected samples and each reaction was carried out in triplicate. *C*t values were determined using the MxPro software. Relative expression between control and infected samples was determined using the 2^-ΔΔ*C*_t_^ method (Livak and Schmittgen, 2001).

### Extraction and Quantification of Salicylic Acid

Salicylic acid extraction was performed on leaf tissue that had been treated with aphids and nematodes both singly and in combination using a modified protocol derived from Raskin et al. (1989). One milliliter of methanol (90%) was added to ground, frozen leaf tissue, and the resulting mixture was vortexed for 1 min followed by sonication in a bath for 5 min. After centrifugation for 5 min at 14,104 × *g*, the supernatant was collected and the pellet was re-extracted with 500 μl methanol (100%), vortexed for 1 min, re-sonicated for 5 min, and re-centrifuged at 14,104 × *g* for a further 5 min. Both supernatants were combined and dried using a GeneVac (EZ-2 series). For free SA quantification the dried samples were re-suspended in 250 μl of 5% trichloroacetic acid (TCA) and vortexed. The sample was extracted twice in cyclohexane and ethyl acetate (1:1), vortexed vigorously and centrifuged at 14,104 × *g* for 1 min. The top organic phase was removed and dried using a GeneVac (EZ-2 series). The remaining phase was subjected to acid hydrolysis using 8M HCl and incubated at 80°C for 1 h to quantify sugar-conjugated (or stored) SA. The sugar-conjugated (or stored) SA sample was extracted twice in cyclohexane and ethyl acetate (1:1), vortexed vigorously and centrifuged at 14,104 × *g* for 1 min. The top organic phase was removed and dried using a GeneVac. The pooled stored SA extract was re-suspended in 600 μl of water and acetonitrile (95:5) and quantified by high-pressure liquid chromatography (HPLC). Analysis was performed using a Supelcosil^TM^ LC-18 column (250 mm × 4.6 mm, 5 μm). An injection volume of 20 μl was separated under isocratic conditions using a mobile phase of water, acetonitrile (HPLC grade) and formic acid (60:40:0.1) at a flow rate of 1 ml/min. SA was detected using a Dionex RF 2000 Fluorescence Detector operated at an emission wavelength of 400 nm and an excitation wavelength of 303 nm, respectively. SA was determined and quantified by comparing peaks of recovered SA using calibration standards. Total SA was calculated as the amount of free SA in plant samples plus the amount of sugar-conjugated (or stored) SA in plant samples. The efficiency of SA recovery was calculated by using a deuterium-labeled internal standard of SA-d_6_. Twelve biological replicates were used for each condition analyzed.

### Jasmonic Acid Quantification

Leaf tissue was harvested as previously described. The samples were ground into a powder in a Tissue Lyser LT (Qiagen, Hilden, Germany) and 1 ml extraction solvent (methanol/H_2_O/formic acid; 80:19:1, v/v/v) was added and mixed. Samples were sonicated at 4°C for 5 min, agitated for 30 min at 4°C and centrifuged at 12,000 × *g* for 10 min at 4°C. The extraction procedure was repeated with 500 μl solvent and the supernatants were combined. JA was analyzed on a UPLC AxION 2 TOF MS system coupled with an Altus SQ Detector (Perkin Elmer, United Kingdom). For the chromatographic separation the solvents were 0.1% HCO_2_H in ultrapure water (A) and 0.1% HCO_2_H in methanol (B), the column was a C18 100 X 1.2 mm (Perkin Elmer, United Kingdom) and the flow rate was set at 0.35 ml min^-1^. The binary analytical gradient used was as follows: 0 min, 1% B; 20 min, 100% B; 22 min, 100% B; 25 min, 1% B. The compound quantification was assured by calibration curve standards in the range of 5–50 ng/ml. The data analysis was performed using Empower 3 software (Waters, United Kingdom).

### Aphid Abundance

To test the effect of *G. pallida* infection on aphids, 10 apterous adults were placed in a 2.5 cm diameter clip cage on a fully expanded, terminal leaf second from the top of a potato plant pre-infected with 10,000 J2 nematodes 14 days previously or mock-inoculated with water. After 24 h all aphids except for five nymphs were removed. The five nymphs were allowed to develop and the number of aphids inside the clip-cage were counted for 8 days to determine the abundance of aphids on nematode-infested plants and non-infected control plants. Five biological replicates for each condition were used in the experiment.

### Data Analysis

The effects of the treatments on gene expression and the levels of endogenous phytohormones JA and SA were determined using a Mann–Whitney *U* test. A Mann–Whitney *U* test was also carried out to compare the abundance of aphids on nematode infected plants against non-infected control plants.

## Results

### Infection of Potato Plants with *Globodera pallida* or *Myzus persicae* Elicits a SA-Mediated Systemic Defense Pathway in the Leaves

**FIGURE 1:**
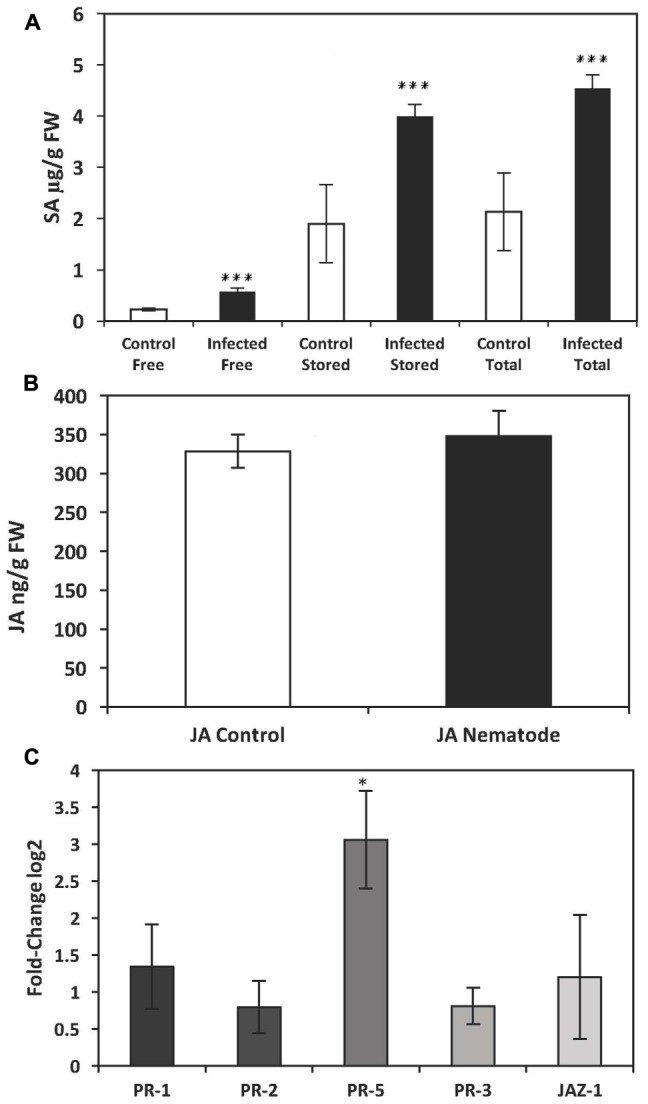
Quantification of endogenous salicylic acid (SA) and jasmonic acid (JA) and analysis of *PR*-gene expression by qRT-PCR in the leaves of potato plants (*Solanum tuberosum* cv. Désirée) infected with the potato cyst nematode, *Globodera pallida.* **(A)** Levels of endogenous SA in leaves of potato plants infected with *G. pallida* 14 days post inoculation (dpi). **(B)** Levels of endogenous JA in leaves of potato plants infected with *G. pallida* 14 dpi. **(C)** Expression levels of *PR*-genes in the leaves of potato plants infected with *G. pallida* at 14 dpi. The presented data are the mean fold changes ± standard errors of biological replicates. The PR transcript levels are relative to uninfected control tissue (baseline set at 0) from different biological replicates [Mann–Whitney *U*, ^∗^*P* < 0.05, ^∗∗∗^*P* < 0.001, *n* = 5 (qPCR and JA analysis), *n* = 12 (endogenous SA)].

There was a significant increase in endogenous SA in the leaves of potato plants 14 days after infection with *G. pallida*. The level of free SA was significantly greater in nematode-infected plants compared to non-infected control plants (mean ± standard error), 571.33 ± 70.09 ng/g FW for infected plants and 231.20 ± 27.21 ng/g FW for control plants (Mann–Whitney *U* = 497.5, *P* = 0.001, sig ≤ 0.05, 2-tailed) (**Figure 1A**). The presence of nematodes also significantly increased total levels of SA in leaves of potato plants, (4541.42 ± 268.2 ng/g FW for nematode-infected plants and 2132.77 ± 758.57 ng/g FW for control plants, *P* ≤ 0.01) (**Figure 1A**). These results suggest an activation of the SAR pathway in the leaves of potato plants, which is mediated by SA (Gaffney et al., 1993).

An elevated level of the endogenous phytohormone SA is known to lead to the expression of pathogen-related (*PR*) genes, some of which are commonly used molecular markers of SAR (Uknes et al., 1993; Bowling et al., 1994; Cao et al., 1994). We therefore measured the expression of *PR-1*, *PR-2*, and *PR-5*, all of which are coordinately regulated by SA (Cao et al., 1994), in nematode-infected plants 14 dpi. Transcripts of all three *PR*-genes were detected in leaf tissue from both infected and non-infected potato plants. However, only the expression of *PR-5* was significantly induced in nematode infected plants (Mann–Whitney *U* = 1.000, *P* = 0.027) (**Figure 1C**). Transcripts of *PR-5*, which encodes a thaumatin-like protein, were approximately three-fold higher in nematode-infested plants relative to control plants (**Figure 1C**).

**FIGURE 2:**
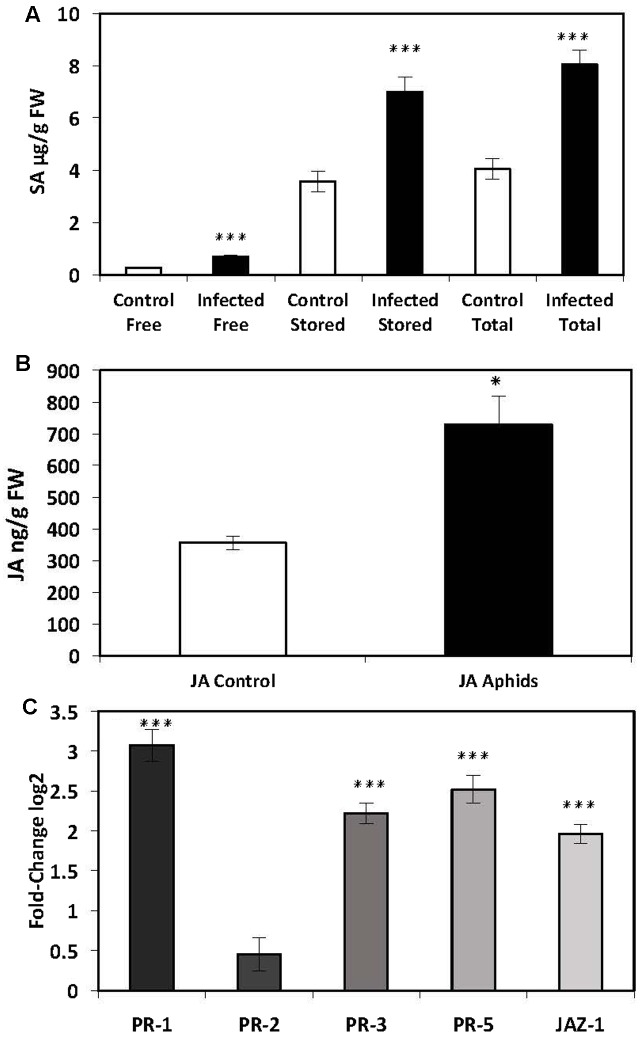
Quantification of endogenous SA and JA and analysis of *PR*-gene expression by qRT-PCR in the leaves of potato plants (*Solanum tuberosum* cv. Désirée) infected with the peach-potato aphid, *Myzus persicae.* **(A)** Levels of endogenous SA in leaves of potato plants infected with *M. persicae* 48 h post inoculation (hpi). **(B)** Levels of endogenous JA in leaves of potato plants infected with *M. persicae* 48 hpi. **(C)** Expression levels of *PR*-genes in the leaves of potato plants infected with *M. persicae* 48 hpi. The presented data are the mean fold changes ± standard errors of biological replicates. The *PR* transcript levels are relative to uninfected control tissue (baseline set at 0) from different biological replicates [Mann–Whitney *U*, ^∗^*P* < 0.05, ^∗∗∗^*P* < 0.001, *n* = 5 (qPCR and JA analysis), *n* = 12 (endogenous SA)].

Five-week-old potato plants infected with aphids were analyzed for endogenous SA and the expression of SA-mediated defense genes. There was a significant increase in free (686 ± 76 ng/g FW, *P* ≤ 0.001), stored (7010 ± 547 ng/g FW, *P* ≤ 0.001) and total (8046 ± 555 ng/g FW, *P* ≤ 0.001) SA in the leaves of potato plants infected with aphids compared to control plants (Free: 276 ± 32 ng/g FW; Stored: 3581 ± 392 ng/g FW; Total: 4055 ± 396 ng/g FW) (**Figure 2A**). The expression of SA-mediated genes *PR*-1 (*P* ≤ 0.001) and *PR*-5 (*P* ≤ 0.001) was also significantly elevated. There was no significant increase in *PR*-2 expression (**Figure 2C**).

### Infection with *Myzus persicae* But Not *Globodera pallida* Elicits a JA-Mediated Systemic Defense Pathway in the Leaves of Potato Plants

In addition to SA-mediated effects, it is well established that JA has an important role in the plant defense pathway. Hence, we also measured endogenous levels of JA as well as transcript levels of *JAZ-1*, which is a nuclear-localized protein involved in jasmonate signaling in addition to *PR-3*. There was a significant increase in endogenous JA in the leaves of plants infected with aphids (729 ± 22 ng/g FW) compared to control plants (356 ± 88 ng/g FW) (*P* ≤ 0.025) (**Figure 2B**). In addition there was a significant increase in transcript levels of *PR*-3 (*P* ≤ 0.001) and *JAZ*-1 (*P* ≤ 0.001) (**Figure 2C**). However, there was no significant increase in endogenous levels of the phytohormone JA in nematode-infected plants 14 dpi (Mann–Whitney *U* = 66.000, *P* = 0.76, sig ≤ 0.05, 2-tailed) (**Figure 1B**) or in the expression of genes involved in the signaling of JA, *PR-*3 (*P* ≤ 0.11) or *JAZ-*1 (*P* ≤ 0.286) (**Figure 1C**) suggesting that nematode infection does not elicit a systemic JA defense response in the leaves of potato plants.

### Co-infection with Both *G. pallida* and *M. persicae* Elicits an Additive SA Defense But a Reduction in the JA Defense Signaling Pathway in the Leaves of Potato Plants

**FIGURE 3:**
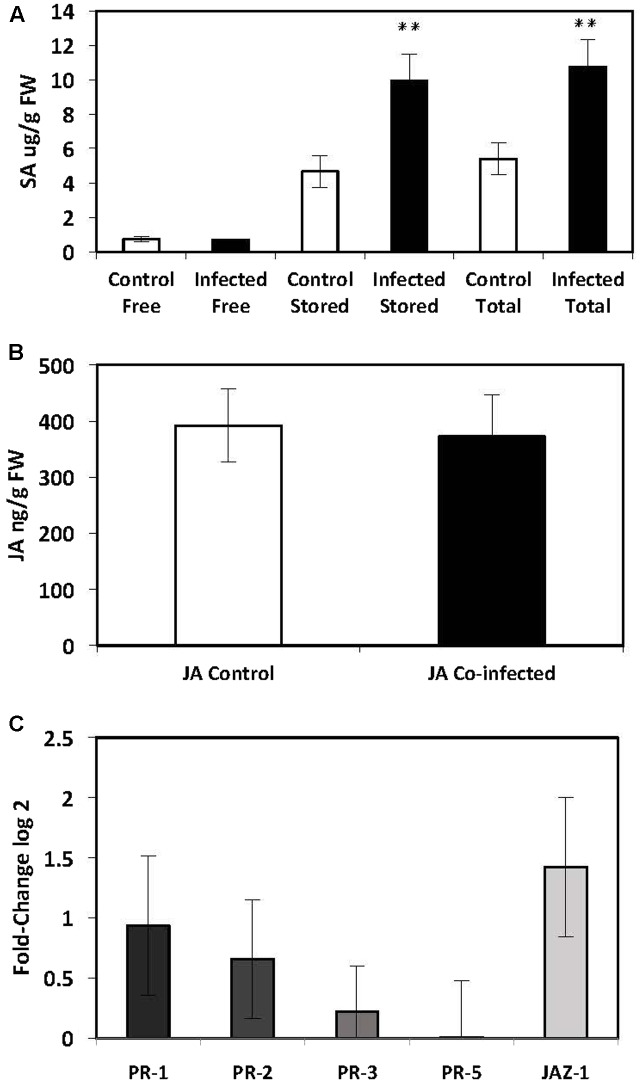
Quantification of endogenous SA and JA and analysis of *PR*-gene expression by qRT-PCR in the leaves of potato plants (*Solanum tuberosum* cv. Désirée) infected with both the potato cyst nematode, *Globodera pallida* and the peach-potato aphid, *Myzus persicae.* **(A)** Levels of endogenous SA in leaves of potato plants infected with *G. pallida* 14 dpi and *M. persicae* 48 hpi. **(B)** Levels of endogenous JA in leaves of potato plants infected with *G. pallida* 14 dpi and M. *persicae* 48 hpi. **(C)** Expression levels of PR-genes in the leaves of potato plants infected with *G. pallida* 14 dpi and *M. persicae* 48 hpi. The presented data are the mean fold changes ± standard errors of biological replicates in qRT-PCR graphs. The *PR* transcript levels are relative to uninfected control tissue (baseline set at 0) from biological replicates [Mann–Whitney *U*, ^∗^*P* < 0.05, ^∗∗^*P* < 0.01, *n* = 5 (qPCR and JA analysis), *n* = 12 (endogenous SA)].

The SA-mediated defence pathway was investigated in the leaves of potato plants that had been infected with both *G. pallida* and *M. persicae*. There was a significant increase in the levels of stored (9943 ± 1522 ng) and total SA (10750 ± 1557 ng) in the leaves of dual infected plants compared to the controls (Stored: 4665 ± 906 ng; Total: 5409 ± 930 ng; *P* ≤ 0.012) (**Figure 3A**). There was no significant difference in the levels of free SA in the leaves of plants that were co-infected (691 ± 45 ng) compared to the controls (743 ± 146 ng) (**Figure 3A**). There was no significant increase in transcript levels of SA-mediated defense genes (**Figure 3A**). The significant increase in the levels of stored SA indicates that the SA-mediated defense pathway is up-regulated in the leaves of potato plants; however, it has not been converted into free SA.

There was no significant change in the level of endogenous JA in plants that had been co-infected with both pests (372 ± 73 ng) compared to the controls (392 ± 64, *P* ≤ 0.855) (**Figure 3B**). Similarly, when the expression of genes involved in the JA signaling pathway were analyzed, there were no significant differences between the leaves of co-infected plants and control plants (**Figure 3C**). Due to a significant increase in endogenous levels of JA and the expression of SA-mediated defenses in the leaves of plants infected with aphids only, the reduction of JA in co-infected plants may indicate an antagonistic suppression of JA by the additive increase in SA caused by both nematode and aphid infection together.

### The Peach-Potato Aphid, *Myzus persicae* Has a Higher Abundance on Potato Plants Pre-infected with *Globodera pallida*

**FIGURE 4:**
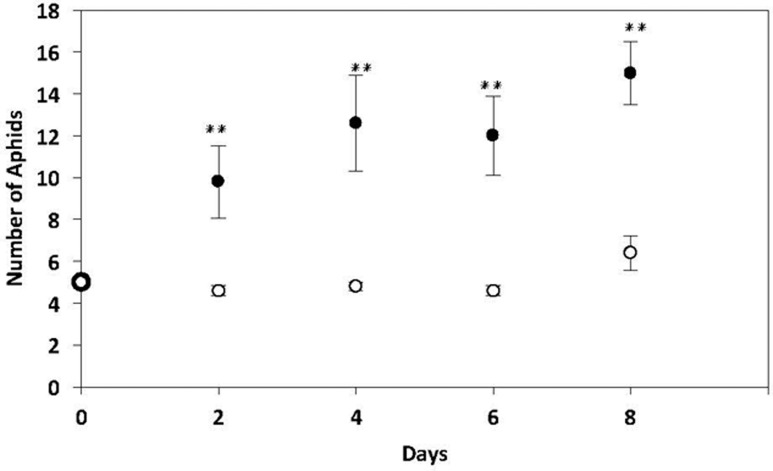
No choice performance assays of *M. persicae* on potato plants pre-infected with 10,000 *G. pallida* J2s for 14 days or non-infected control potato plants. Black dots represent aphids present on plant pre-infected with nematodes. White dots represent aphids present on non-infected control plants. There were more *M. persicae* present on nematode-infested plants from day 2 to day 8 compared to non-infected control plants (*n* = 5, ^∗∗^*P* < 0.01).

There was a significant increase in the abundance of aphids reared on potato plants pre-infected with nematodes for 14 days compared with aphids reared on non-infected control plants (Mann–Whitney *U* = 3.000, *P* = 0.011, sig ≤ 0.05, 2-tailed) (**Figure 4**).

## Discussion

Our results show how the molecular and biochemical response of the potato plant to attack by a below-ground pathogen, in this case plant-parasitic nematodes, can indirectly influence herbivore populations above ground through systemic changes in endogenous phytohormones and expression of associated genes.

### Plant Responses to Cyst Nematode and Aphid Infection Singly and in Combination

Previous studies have revealed that defense signaling pathways are involved in compatible interactions of plants with cyst nematodes (*Heterodera* and *Globodera* spp.) (Jammes et al., 2005; Ithal et al., 2007; Wubben et al., 2008). Similarly, it is well known that many plant defense signaling pathways are up-regulated in response to aphid feeding (De Vos et al., 2005; Kuśnierczyk et al., 2008; Broekgaarden et al., 2011). Our analysis has shown that expression of *PR-5*, a molecular marker commonly used to indicate activation of SAR (Uknes et al., 1993; Bowling et al., 1994), was significantly increased in leaves of potato plants following infection with *G. pallida* for 14 days and also in the leaves of 5-week-old plants infected with *M. persicae* for 48 h. This correlates with the significant increase in free and total SA in leaves of potato plants: the accumulation of the phytohormone SA is required for the activation of SAR in distal tissues of the infected plant (Gaffney et al., 1993). Taken together these results indicate activation of a SAR-induced potato defense pathway following parasitism by *G. pallida* and infection with *M. persicae* singly. There was no significant increase in the expression *PR*-1 or *PR-*2 in the leaves of nematode-infected potato plants at the time-point examined. Expression of the orthologous genes was reported to increase in the leaves of *Arabidopsis thaliana* in response to cyst nematode infection, however, this increase was transient and varied considerably between investigations (Wubben et al., 2008; Hamamouch et al., 2011). The length of time post-infection, together with the initial nematode burden, may be critical in determining if *PR*-gene induction is observed. It is well documented that there is mutual antagonism between SA and JA signaling pathways (Pieterse et al., 2012), therefore the phytohormone JA and the expression levels of the JA-dependent associated genes *PR-3* and *JAZ-1*, a nuclear-localized protein involved in jasmonate signaling (Thines et al., 2007) were quantified. No significant differences were found between nematode-infected plants and control plants in either the amount of JA or the expression of *PR-3* and *JAZ*-1, suggesting that infection with the potato cyst nematode does not alter the JA signaling pathway in the potato plant at 14 dpi. Alternatively, this could indicate antagonistic cross-talk between the SA and JA pathways following infection with *G. pallida*, as both endogenous SA and the expression of *PR-5* was significantly up-regulated. In contrast, it was found that aphid infection induced the JA signaling pathway in the leaves of potato plants as both JA and the expression of *PR*-3 and *JAZ*-1 were significantly up-regulated compared to control plants.

Co-infection of the potato with both *G. pallida* and *M. persicae* had a different and unique impact on the levels of endogenous phytohormones and expression of defense-related genes compared to plants that had been infected with each pest singly. An additive effect on SA was observed in co-infected plants, an effect that may be assumed when two pests are applied to a plant. However, a reduced JA effect was noted in dual infected plants even though JA was present in the leaves of plants infected with aphids in isolation. There is literature to suggest that phytohormones do not act independently of one another. The interaction between SA and JA is complex with the main interaction between these two pathways being mutual antagonism (Kunkel and Brooks, 2002). SA has been shown to have an inhibitory effect on JA in tomato (Doherty et al., 1988; Pena-Cortés et al., 1993) and in *Arabidopsis* (Clarke et al., 2000; Gupta et al., 2000). Therefore, a lack of JA in the leaves of co-infected plants could be construed as antagonistic crosstalk because although infection with plant-parasitic nematodes did not elicit the JA defense pathway in potato plants, infection with aphids alone did.

### Herbivore Responses to Plant Parasitic Nematode Infection

Plant-mediated interactions between plant parasitic nematodes and aerial pests studied to date have been variable: susceptibility to shoot pathogens and resistance to phloem feeders have been reported with the outcome depending on the parasitic strategy of the nematode involved in the interaction (Biere and Goverse, 2016). To the best of our knowledge there have been no studies of plant-mediated interactions between the potato cyst nematode and specialized above-ground pests or pathogens of potato, however, there have been reports of interactions between *G. rostochiensis* and below-ground pathogens such as the soil-borne fungus of potato, *Rhizoctonia solani* (Back et al., 2006). A reduced aphid performance was reported when *Plantago lanceolata* (Wurst and van der Putten, 2007) was infected with the migratory nematode, *Pratylenchus penetrans*. Similarly, a decrease in the fecundity of aphids was observed when *Agrostis capillaris* was infected with a mixture consisting of ectoparasites and migratory endoparasites (Bezemer et al., 2003). Reports using sedentary endoparasites have found negative or neutral impacts on aphids. An infection of *H. schachtii* on *B. oleracea* resulted in reduced growth and fecundity of a specialist aphid species, *Brevicoryne brassicae* as well as a generalist species, *M. persicae* (Hol et al., 2013). However, in another study using a mix of different parasitic nematode species, no effect on the performance of *B. brassicae* was found (Kabouw et al., 2011). Our observation that *G. pallida*, a sedentary endoparasitic nematode, indirectly and positively influences the abundance of *M. persicae* highlights how aphids may be more damaging to the potato crop in areas where *G. pallida* is present compared to such areas where there is no infection, however, this requires further investigation. Our study is in contrast to these previous studies and to our knowledge is the first to report the combined molecular and biochemical response of the potato to nematode infection.

Systemic plant resistance to insect herbivores is mediated by the SA and JA wound signaling pathways and the, usually antagonistic, crosstalk between them (Pieterse et al., 2012; Stam et al., 2014). In addition to their role in regulating resistance to biotrophic pathogens, SA-mediated defensive pathways are known to be induced by phloem-feeding insects, and there have also been reports suggesting that SA itself is an effective chemical defense against phloem-sucking herbivory animals (Kaloshian and Walling, 2005; Donovan et al., 2013). As expected, we found induction of the SA pathway in response to nematodes, but any adverse effects of this on the aphids are likely to be negated by the benefits of SA-mediated reductions of the JA-mediated pathway responsible for plant resistance to herbivores (Lazebnik et al., 2014). Indeed, aphids are believed to circumvent the plant’s immune system by eliciting the SA signaling pathway in order to antagonize and suppress the JA one, which is important in mediating resistance to phloem feeders (Ellis et al., 2002; Zhu-Salzman et al., 2004). Thus, our observation of more aphids present on nematode infested plants could reflect circumvention of the SA-mediated defense pathway of the potato plant by *M. persicae*. Our analysis of the JA-mediated defense pathway in the potato plant showed no up-regulation of endogenous JA or expression of *PR-3* or *JAZ-1* in leaves of potato plants infected with nematodes when compared to control plants. Aphids could benefit from the situation in which the hormone has not been elicited or even suppressed.

## Conclusion

Our biochemical and molecular data reveal the potential mechanisms underpinning a positive asymmetric interaction between a sedentary endoparasitic nematode and a sap-sucking insect. The SA pathway and PR defense gene expression is altered in the potato plant following infection with *G. pallida* and these changes indirectly influence the performance of the peach potato aphid *M. persicae.* Our study highlights how multiple stresses elicit a unique molecular and biochemical response compared to singly stressed plants. It also demonstrates the importance of analysing hormonal crosstalk when seeking to understand plant defensive responses to co-incident attack by pests and pathogens.

## Author Contributions

Designed research: GH, CL, MD, SH, and PU. Performed research: GH. Analyzed the data: GH, KJF, and MD. Wrote the manuscript: GH, CL, KJF, MD, SH, and PU.

## Conflict of Interest Statement

The authors declare that the research was conducted in the absence of any commercial or financial relationships that could be construed as a potential conflict of interest.

## Footnotes

1 www.ncbi.nlm.nih.gov

2 http://primer3.ut.ee/

## References

[B1] Alkharouf N. W. Klink V. P. Chouikha I. M. Beard H. S. MacDonald M. Meyer S. (2006). Timecourse microarray analyses reveal global changes in gene expression of susceptible *Glycine max* (soybean) roots during infection by *Heterodera glycines* (soybean cyst nematode). *Planta* 224 838–852. 10.1007/s00425-006-0270-8 16575592

[B2] Atkinson N. J. Jain R. Urwin P. E. (2015). “The response of plants to simultaneous biotic and abiotic stress,” in *Combined Stresses in Plants*, ed. Mahalingam R. (Berlin: Springer).

[B3] Back M. Haydock P. Jenkinson P. (2006). Interactions between the potato cyst nematode *Globodera rostochiensis* and diseases caused by *Rhizoctonia solani* AG3 in potatoes under field conditions. *Eur. J. Plant Pathol.* 114 215–223. 10.1007/s10658-005-5281-y

[B4] Bezemer T. M. Wagenaar R. van Dam N. Wäckers F. (2003). Interactions between above- and belowground insect herbivores as mediated by the plant defence system. *Oikos* 101 555–562. 10.1034/j.1600-0706.2003.12424.x

[B5] Biere A. Goverse A. (2016). Plant-mediated systemic interactions between pathogens, parasitic nematodes, and herbivores above- and belowground. *Annu. Rev. Phytopathol.* 54 499–527. 10.1146/annurev-phyto-080615-100245 27359367

[B6] Bowling S. A. Guo A. Cao H. Gordon A. S. Klessig D. F. Dong X. (1994). A mutation in Arabidopsis that leads to constitutive expression of systemic acquired resistance. *Plant Cell* 6 1845–1857. 10.1105/tpc.6.12.1845 7866028 PMC160566

[B7] Broekgaarden C. Voorrips R. E. Dicke M. Vosman B. (2011). Transcriptional responses of *Brassica nigra* to feeding by specialist insects of different feeding guilds. *Insect Sci.* 18 259–272. 10.1111/j.1744-7917.2010.01368.x

[B8] Cao H. Bowling S. A. Gordon A. S. Dong X. (1994). Characterisation of an Arabidopsis mutant that is nonresponsive to inducers of systemic acquired resistance. *Plant Cell* 6 1583–1592. 10.1105/tpc.6.11.1583 12244227 PMC160545

[B9] Clarke J. D. Volko S. M. Ledford H. Ausubel F. M. Dong X. (2000). Roles of salicylic acid, jasmonic acid, ethylene in cpr-induced resistance in Arabidopsis. *Plant Cell* 12 2175–2190. 10.1105/tpc.12.11.2175 11090217 PMC150166

[B10] De Vos M. van Oosten V. R. van Poecke R. M. van Pelt J. A. Pozo M. J. Mueller M. J. (2005). Signal signature and transcriptome changes of *Arabidopsis* during pathogen and insect attack. *Mol. Plant Microbe Interact.* 18 923–937. 10.1094/MPMI-18-0923 16167763

[B11] Denancé N. Sánchez-Vallet A. Goffner D. Molina A. (2013). Disease resistance or growth: the role of plant hormones in balancing immune responses and fitness costs. *Front. Plant Sci.* 4:155. 10.3389/fpls.2013.00155 23745126 PMC3662895

[B12] Dicke M. van Loon J. J. Soler R. (2009). Chemical complexity of volatiles from plants induced by multiple attack. *Nat. Chem. Biol.* 5 317–324. 10.1038/nchembio.169 19377458

[B13] Dixon A. F. G. Kindlmann P. (1998). “Population dynamics of aphids,” in *Insect Populations in Theory and in Practice*, eds Dempster J. P. McLean I. F. G. (Dordrecht: Kluwer), 207–230. 10.1007/978-94-011-4914-3_9

[B14] Doherty H. M. Selvendran R. R. Bowles D. J. (1988). The wound response of tomato plants can be inhibited by aspirin and related hydroxyl-benzoic acids. *Physiol. Mol. Plant Pathol.* 33 377–384. 10.1016/0885-5765(88)90004-5

[B15] Donovan M. P. Nabity P. D. DeLucia E. H. (2013). Salicylic acid-mediated reductions in yield in *Nicotiana attenuata* challenged by aphid herbivory. *Arthropod Plant Interact.* 7 45–52. 10.1007/s11829-012-9220-5

[B16] Ellis C. Karafyllidis I. Turner J. G. (2002). Constitutive activation of jasmonate signalling in an *Arabidopsis* mutant correlates with enhanced resistance to *Erysiphe cichoracearum*, *Pseudomonas syringae*, and *Myzus persicae*. *Mol. Plant Microbe Interact.* 15 1025–1030. 10.1094/MPMI.2002.15.10.1025 12437300

[B17] Erb M. Robert C. A. Hibbard B. E. Turlings T. C. (2011). Sequence of arrival determines plant-mediated interactions between herbivores. *J. Ecol.* 99 7–15. 10.1111/j.1365-2745.2010.01757.x

[B18] Fenwick D. W. (1940). Methods for the recovery and counting of cysts of *Heterodera schachtii* from soil. *J. Helminthol.* 18 155–172. 10.1017/S0022149X00031485

[B19] Fidantsef A. L. Stout M. J. Thaler J. Duffey S. Bostock R. (1999). Signal interactions in pathogen and insect attack: expression of lipoxygenase, proteinase inhibitor II, and pathogenesis-related protein P4 in the tomato, *Lycopersicon esculentum*. *Physiol. Mol. Plant Pathol.* 54 97–114. 10.1006/pmpp.1998.0192

[B20] Foster S. P. Denholm I. Devonshire A. L. (2000). The ups and downs of insecticide resistance in peach-potato aphids, *Myzus persicae* in the UK. *Crop Protect.* 19 873–879. 10.1016/S0261-2194(00)00115-0

[B21] Gaffney T. Friedrich L. Vernooji B. Negrotto D. Nye G. Uknes S. (1993). Requirement of salicylic acid for the induction of systemic acquired resistance. *Science* 261 754–754. 10.1126/science.261.5122.754 17757215

[B22] Glazebrook J. (2005). Contrasting mechanisms of defence against biotrophic and necrotrophic pathogens. *Annu. Rev. Phytopathol.* 43 205–227. 10.1146/annurev.phyto.43.040204.135923 16078883

[B23] Gupta V. Willits M. G. Glazebrook J. (2000). *Arabidopsis thaliana* EDS4 contributes to salicylic acid (SA)-dependent expression of defence responses: evidence for inhibition of jasmonic acid signalling by SA. *Mol. Plant Microbe Interact.* 13 503–511. 10.1094/MPMI.2000.13.5.503 10796016

[B24] Hamamouch N. Li C. Seo P. J. Park C.-M. Davis E. L. (2011). Expression of Arabidopsis pathogenesis-related genes during nematode infection. *Mol. Plant Pathol.* 12 355–364. 10.1111/j.1364-3703.2010.00675.x 21453430 PMC6640486

[B25] Heungens K. Mugniéry D. van Montagu M. Gheysen G. Niebel A. (1996). A method to obtain disinfected *Globodera* infective juveniles directly from cysts. *Fundament. Appl. Nematol.* 19 91–93.

[B26] Hofmann J. El Ashry A. E. N. Anwar S. Erban A. Kopka J. Grundler F. (2010). Metabolic profiling reveals local and systemic responses of host plants to nematode parasitism. *Plant J.* 62 1058–1071. 10.1111/j.1365-313X.2010.04217.x 20374527 PMC2904900

[B27] Hol W. G. De Boer W. Termorshuizen A. J. Meyer K. M. Schneider J. H. van der Putten W. H. (2013). *Heterodera schachtii* nematodes interfere with aphid-plant relations on *Brassica oleracea.* *J. Chem. Ecol.* 39 1193–1203. 10.1007/s10886-013-0338-4 24014097 PMC3790247

[B28] Hong S. C. Donaldson J. Gratton C. (2010). Soybean cyst nematode effects on soybean aphid preference and performance in the laboratory. *Environ. Entomol.* 39 1561–1569. 10.1603/EN10091 22546453

[B29] Huang W. Robert C. A. Hervé M. R. Hu L. Bont Z. Erb M. (2016). A mechanism for sequence specificity in plant-mediated interactions between herbivores. *New Phytol.* 214 169–179. 10.1111/nph.14328 27898177 PMC6079637

[B30] Ithal N. Recknor J. Nettleton D. Hearne L. Maier T. Baum T. J. (2007). Parallel genome-wide expression profiling of host and pathogen during soybean cyst nematode infection of soybean. *Mol. Plant Microbe Interact.* 20 293–305. 10.1094/MPMI-20-3-0293 17378432

[B31] Jammes F. Lecomte P. Almeida-Engler J. Bitton F. Martin-Magniette M. L. Renou J. P. (2005). Genome-wide expression profiling of the host response to root-knot nematode infection in Arabidopsis. *Plant J.* 44 447–458. 10.1111/j.1365-313X.2005.02532.x 16236154

[B32] Johnson S. N. Clark K. E. Hartley S. E. Jones T. H. McKenzie S. W. Koricheva J. (2012). Aboveground-belowground herbivore interactions: a meta-analysis. *Ecology* 93 2208–2215. 10.1890/11-2272.1 23185882

[B33] Kabouw P. Kos M. Kleine S. Vokenhuber E. A. Van Loon J. J. A. Van der Putten W. H. (2011). Effects of soil organisms on aboveground multitrophic interactions are consistent between plant genotypes mediating the interaction. *Entomol. Exp. Appl.* 139 197–206. 10.1111/j.1570-7458.2011.01123.x

[B34] Kaloshian I. Walling L. (2005). Hemipterans as plant pathogens. *Annu. Rev. Phytopathol.* 43 491–521. 10.1146/annurev.phyto.43.040204.135944 16078893

[B35] Kaplan I. Halitschke R. Kessler A. Rehill B. J. Sardanelli S. Denno R. F. (2008). Physiological integration of roots and shoots in plant defence strategies link above- and belowground herbivory. *Ecol. Lett.* 11 841–851. 10.1111/j.1461-0248.2008.01200.x 18479456

[B36] Kasprowicz L. Malloch G. Pickup J. Fenton B. (2008). Spatial and temporal dynamics of *Myzus persicae* clones in fields and suction traps. *Agric. For. Entomol.* 10 91–100. 10.1111/j.1461-9563.2008.00365.x

[B37] Kissoudis C. van de Wiel C. Visser R. G. van der Linden G. (2014). Enhancing crop resilience to combined abiotic and biotic stress through the dissection of physiological and molecular crosstalk. *Front. Plant Sci.* 5:207. 10.3389/fpls.2014.00207 24904607 PMC4032886

[B38] Kolbe W. (1970). Influence of direct feeding damage on yields of heavily aphid-infested potato crops. *Pflanzens. Nachr. Bayer* 23 273–282.

[B39] Kombrink E. Schröder M. Hahlbrock K. (1988). Several “pathogenesis-related” proteins in potato are 1, 3-β-glucanases and chitinases. *Proc. Natl. Acad. Sci. U.S.A.* 85 782–786. 10.1073/pnas.85.3.782 16578829 PMC279639

[B40] Kunkel B. N. Brooks D. M. (2002). Crosstalk between signalling pathways in pathogen defence. *Curr. Opin. Plant Biol.* 5 325–331. 10.1016/S1369-5266(02)00275-3 12179966

[B41] Kuśnierczyk A. Winge P. Jørstad T. S. Troczynska J. Rossiter J. T. Bones A. M. (2008). Towards global understanding of plant defence against aphids-timing and dynamics of early Arabidopsis defence responses to cabbage aphid (*Brevicoryne brassicae*) attack. *Plant Cell Environ.* 31 1097–1115. 10.1111/j.1365-3040.2008.01823.x 18433442

[B42] Kutyniok M. Müller C. (2012). Crosstalk between above- and belowground herbivores is mediated by minute metabolic responses of the host *Arabidopsis thaliana*. *J. Exp Bot.* 63 6199–6210. 10.1093/jxb/ers274 23045608 PMC3481212

[B43] Kutyniok M. Persicke M. Müller C. (2014). Effects of root herbivory by nematodes on the performance and preference of a leaf-infesting generalist aphid depend on nitrate fertilisation. *J. Chem. Ecol.* 40 118–127. 10.1007/s10886-014-0387-3 24500735

[B44] Lazebnik J. Frago E. Dicke M. van Loon J. J. (2014). Phytohormone mediation of interactions between herbivores and plant pathogens. *J. Chem. Ecol.* 40 730–741. 10.1007/s10886-014-0480-7 25059974

[B45] Lilley C. J. Atkinson H. J. Urwin P. E. (2005). Molecular aspects of cyst nematodes. *Mol. Plant Pathol.* 6 577–588. 10.1111/j.1364-3703.2005.00306.x 20565681

[B46] Livak K. J. Schmittgen T. D. (2001). Analysis of relative gene expression data using real-time quantitative PCR and the 2^-ΔΔ*C*_T_^ method. *Methods* 25 402–408. 10.1006/meth.2001.1262 11846609

[B47] Loake G. Grant M. (2007). Salicylic acid in plant defence – the players and protagonists. *Curr. Opin. Plant Biol.* 10 466–472. 10.1016/j.pbi.2007.08.008 17904410

[B48] Masters G. J. Hefin Jones T. Rogers M. (2001). Host-plant mediated effects of root herbivory on insect seed predators and their parasitoids. *Oecologia* 127 246–250. 10.1007/s004420000569 24577656

[B49] Matton D. P. Brisson N. (1989). Cloning, expression and sequence conservation of pathogenesis-related gene transcripts of potato. *Mol. Plant Microbe Interact.* 2 325–331. 10.1094/MPMI-2-325 2520162

[B50] Miller G. Suzuki N. Ciftci-Yilmaz S. Mittler R. (2010). Reactive oxygen species homeostasis and signalling during drought and salinity stress. *Plant Cell Environ.* 33 453–467. 10.1111/j.1365-3040.2009.02041.x 19712065

[B51] Minnis S. Haydock P. P. J. Ibrahim S. Grove I. Evans K. Russell M. (2002). Potato cyst nematodes in England and Wales – occurrence and distribution. *Ann. Appl. Biol.* 140 187–195. 10.1111/j.1744-7348.2002.tb00172.x 11721525

[B52] Nicot N. Hausman J. F. Hoffmann L. Evers D. (2005). Housekeeping gene selection for real-time RT-PCR normalisation in potato during biotic and abiotic stress. *J. Exp. Biol.* 56 2907–2914. 10.1093/jxb/eri285 16188960

[B53] Papadopoulou G. V. van Dam N. M. (2017). Mechanisms and ecological implications of plant-mediated interactions between belowground and aboveground insect herbivores. *Ecol. Res.* 32 13–26. 10.1007/s11284-016-1410-7

[B54] Peleg Z. Blumwald E. (2011). Hormone balance and abiotic stress tolerance in crop plants. *Curr. Opin. Plant Biol.* 14 290–295. 10.1016/j.pbi.2011.02.001 21377404

[B55] Pena-Cortés H. Albrecht T. Prat S. Weiler E. W. Willmitzer L. (1993). Aspirin prevents wound-induced gene expression in tomato leaves by blocking jasmonic acid biosynthesis. *Planta* 191 123–128. 10.1007/BF00240903

[B56] Pieterse C. M. J. van der Does D. Zamioudis C. Leon-Ryas A. van Wees S. C. M. (2012). Hormonal modulation of plant immunity. *Annu. Rev. Cell Dev. Biol.* 28 489–521. 10.1146/annurev-cellbio-092910-154055 22559264

[B57] Pollard D. G. (1973). Plant penetration by feeding aphids (Hemiptera, Aphidoidea): a review. *Bull. Entomol. Res.* 62 631–714. 10.1017/S0007485300005526

[B58] Radcliffe E. B. (1982). Insect pests of potato. *Annu. Rev. Entomol.* 27 173–204. 10.1146/annurev.en.27.010182.001133

[B59] Raskin I. Turner I. M. Melander W. R. (1989). Regulation of heat production in the inflorescences of an Arum lily by endogenous salicylic acid. *Proc. Natl. Acad. Sci. U.S.A.* 86 2214–2218. 10.1073/pnas.86.7.2214 16594020 PMC286882

[B60] Reiss E. Horstmann C. (2001). Drechslera teres-infected barley (*Hordeum vulgare* L.) leaves accumulate eight isoforms of thaumatin-like proteins. *Physiol. Mol. Plant Pathol.* 58 183–188. 10.1006/pmpp.2001.0325

[B61] Schoonhoven L. M. van Loon J. J. A. Dicke M. (2005). *Insect-Plant Biology.* Oxford: Oxford University Press.

[B62] Stam J. M. Kroes A. Li Y. Gols R. van Loon J. J. Poelman E. H. (2014). Plant interactions with multiple insect herbivores: from community to genes. *Plant Biol.* 65 689–713. 10.1146/annurev-arplant-050213-035937 24313843

[B63] Szakasits D. Heinen P. Wieczorek K. Hofmann J. Wagner F. Kreil D. P. (2009). The transcriptome of syncytia induced by the cyst nematode *Heterodera schachtii* in Arabidopsis roots. *Plant J.* 57 771–784. 10.1111/j.1365-313X.2008.03727.x 18980640 PMC2667683

[B64] Thines B. Katsir L. Melotto M. Niu Y. Mandaokar A. Liu G. (2007). JAZ repressor proteins are targets of the SCF (COI1) complex during jasmonate signalling. *Nature* 448 661–665. 10.1038/nature05960 17637677

[B65] Ton J. Flors V. Mauch-Mani B. (2009). The multifaceted role of ABA in disease resistance. *Trends Plant Sci.* 14 310–317. 10.1016/j.tplants.2009.03.006 19443266

[B66] Trudgill D. L. (1986). Yield losses caused by potato cyst nematodes: a review of the current position in Britain and prospects for improvements. *Ann. Appl. Biol.* 108 181–198. 10.1111/j.1744-7348.1986.tb01979.x

[B67] Uehara T. Sugiyama S. Matsuura H. Arie T. Masuta C. (2010). Resistant and susceptible responses in tomato to cyst nematode are differentially regulated by salicylic acid. *Plant Cell Physiol.* 51 1524–1536. 10.1093/pcp/pcq109 20660227

[B68] Uknes S. Dincher S. Friedrich L. Negrotto D. Williams S. Thompson-Taylor H. (1993). Regulation of pathogenesis-related protein-1a gene expression in tobacco. *Plant Cell* 5 159–169. 10.1105/tpc.5.2.159 8453300 PMC160259

[B69] van Dam N. M. Heil M. (2011). Multitrophic interactions below and above ground: en route to the next level. *J. Ecol.* 99 77–88. 10.1111/j.1365-2745.2010.01761.x

[B70] van Dam N. M. Raaijmakers C. E. van der Putten W. H. (2005). Root herbivory reduces growth and survival of the shoot feeding specialist *Pieris rapae* on *Brassica nigra*. *Entomol. Exp. Appl.* 115 161–170. 10.1111/j.1570-7458.2005.00241.x

[B71] Van Emden H. F. Eastop V. F. Hughes R. D. Way M. J. (1969). The ecology of *Myzus persicae*. *Annu. Rev. Entomol.* 14 197–270. 10.1146/annurev.en.14.010169.001213

[B72] van Geem M. Gols R. Raaijmakers C. E. Harvey J. A. (2016). Effects of population-related variation in plant primary and secondary metabolites on aboveground and belowground multitrophic interactions. *Chemoecology* 26 219–233. 10.1007/s00049-016-0222-0 27795618 PMC5063910

[B73] Vandegehuchte M. L. De Le Peña E. Bonte D. (2010). Interactions between root and shoot herbivores of *Ammophila arenaria* in the laboratory does not translate into correlated abundances in the field. *Oikos* 119 1011–1019. 10.1111/j.1600-0706.2009.18360.x

[B74] Wang B. Liu J. Tian Z. Song B. Xie C. (2005). Monitoring the expression patterns of potato genes associated with quantitative resistance to late blight during *Phytophthora infestans* infection using cDNA microarrays. *Plant Sci.* 169 1155–1167. 10.1016/j.plantsci.2005.07.020

[B75] Wardle D. A. Bardgett R. D. Klironomos J. N. Setälä H. van der Putten W. H. Wall D. H. (2004). Ecological linkages between aboveground and belowground biota. *Science* 304 1629–1633. 10.1126/science.1094875 15192218

[B76] Wubben M. J. E. Jin J. Baum T. J. (2008). Cyst nematode parasitism of *Arabidopsis thaliana* is inhibited by salicylic acid (SA) and elicits uncoupled SA-independent pathogenesis-related gene expression in roots. *Mol. Plant Microbe Interact.* 21 424–432. 10.1094/MPMI-21-4-0424 18321188

[B77] Wurst S. van der Putten W. H. (2007). Root herbivore identity matters in plant-mediated interactions between root and shoot herbivores. *Basic Appl. Biol.* 8 491–499. 10.1016/j.baae.2006.09.015

[B78] Zhu-Salzman K. Salzman R. A. Ahn J. E. Koiwa H. (2004). Transcriptional regulation of sorghum defence determinants against a phloem-feeding aphid. *Plant Physiol.* 134 420–431. 10.1104/pp.103.028324 14701914 PMC316321

