# Supplementary material for: A Plant-Feeding Nematode Indirectly Increases the Fitness of an Aphid
Source: Front Plant Sci. 2017 Nov 3;8:1897. doi: 10.3389/fpls.2017.01897 (PMC5701616; doi:10.3389/fpls.2017.01897)
Supplement: Supplementary file 1 [file Table_1.PDF]

Supplementary Data Table 1. Sequence and mean PCR efficiency of primers used for RT-qPCR analysis

Gene Name	Accession Number	Orientation of Primer	Primer Sequences (5'-3')	Mean PCR Efficiency
Elongation Factor 1-α	AB061263	Forward	ATTGGAAACGGATATGCTCCA	96.5%
		Reverse	TCCTTACCTGAACGCCTGTCA	
PR-1	AJ250136.1	Forward	TGGTACCAACCAATGTGCAA	98.5%
		Reverse	AATGAACCACCATCCGTTGT	
PR-2	U01901	Forward	CTGTTTATGCTGCGATGGAA	94.4%
		Reverse	GTCTTGTGTGGCACCAAATG	
PR-5	AY737315.1	Forward	GCATCTGGGCATCTTTGTTT	93.7%
		Reverse	GCACGGTTTTCAAGACGAAT	
JAZ-1	EF591123	Forward	AGCCAACAAACAGAACCCCA	101%
		Reverse	TCACACCAGATTGATCAGCTGT	
